# Digitally Guided Frontal Sinus Fracture Fixation: A Point-of-Care “In-House” Biomodel Protocol with Cyanoacrylate-Assisted Fragment Stabilization

**DOI:** 10.3390/jcm15052057

**Published:** 2026-03-08

**Authors:** Manuel Tousidonis, Saad Khayat, Cristina Maza-Muela, Rocio Franco-Herrera, Ruben Pérez-Mañanes, Jose-Antonio Calvo-Haro, Maria J. Troulis, Carlos Navarro-Cuellar, Jose-Ignacio Salmeron, Santiago Ochandiano

**Affiliations:** 1 Department of Oral and Maxillofacial Surgery, Gregorio Marañon University Hospital, 28007 Madrid, Spain; 2 Gregorio Marañón Research Institute, 28007 Madrid, Spain; 3 Advanced Planning and 3D Manufacturing Unit (UPAM3D), Hospital General Universitario Gregorio Marañón, Dr. Esquerdo 46, 28007 Madrid, Spain; 4 Department of Surgery, Gregorio Marañón University Hospital, 28007 Madrid, Spain; 5 Surgery Department, Faculty of Medicine, Universidad Complutense de Madrid, 28040 Madrid, Spain; 6 Oral and Maxillofacial Surgery, Department of Surgery, Beth Israel Deaconess Medical Center, Boston, MA 02215, USA; 7 Harvard School of Dental Medicine and Harvard Medical School, Boston, MA 02115, USA

**Keywords:** biomodel-guided reduction, frontal sinus fracture, 3D-printed template, extracorporeal reduction, surgical adhesive, cyanoacrylate-assisted fixation, point-of-care 3D printing, in-house manufacturing, titanium microplates, aesthetic contour restoration

## Abstract

**Background/Objectives:** Frontal sinus fractures are uncommon injuries that may cause persistent aesthetic deformity when the anterior wall is comminuted, as small irregular fragments are difficult to stabilize with conventional osteosynthesis alone. **Methods:** We describe a point-of-care digital workflow combining 3D planning/printing and cyanoacrylate-assisted fixation for an isolated comminuted anterior frontal sinus wall fracture. A young adult presented with a depressed forehead contour after assault; computed tomography confirmed at least four displaced fragments. **Results:** A two-part 3D-printed biomodel was manufactured in-house to visualize the defect and guide extracorporeal reconstruction. Through a coronal approach, fragments were mobilized and anatomically reassembled using the biomodel as a reference; sinonasal drainage was preserved and sinus obliteration was not required. Because fragment size and geometry limited screw purchase, a modified *N*-butyl-2-cyanoacrylate adhesive (Glubran 2) was applied as an adjunct to maintain reduction, followed by reinforcement with titanium microplates. Postoperative recovery was uneventful, with immediate restoration of forehead contour and no early complications; postoperative imaging confirmed satisfactory alignment. **Conclusions:** This case supports the feasibility of integrating point-of-care 3D biomodeling with cyanoacrylate as a coadjuvant to microplate fixation in selected comminuted frontal sinus fractures to enhance fragment handling and contour restoration.

## 1. Introduction

Frontal sinus fractures are relatively uncommon injuries within cranio-maxillofacial trauma, yet they remain clinically relevant because of their potential for both early intracranial complications and delayed sinonasal sequelae. Management is guided by fracture pattern and displacement, involvement of the anterior and/or posterior table, the status of the nasofrontal outflow tract (NFOT), and the presence or persistence of cerebrospinal fluid (CSF) leak [1,2,3,4]. These variables determine whether observation is appropriate or whether operative strategies such as open reduction and internal fixation (ORIF), sinus obliteration, or cranialization are indicated [1,3,4]. Importantly, long-term complications—particularly mucocele/mucopyocele, chronic infection, osteomyelitis, and intracranial abscess—may appear years after the initial injury or intervention, supporting careful patient selection and long-term follow-up [1,2].

Over the last decade, several institutional algorithms and contemporary reviews have increasingly questioned the need for radical cranial procedures in all displaced fractures, emphasizing a more conservative trend when posterior table integrity is preserved, NFOT patency is likely, and CSF leakage is absent or resolves [2,3,4]. Decision-making has therefore evolved toward individualized treatment, balancing complication prevention with minimizing surgical morbidity [2,3,4].

While posterior table injuries primarily raise concerns about intracranial communication and infection risk, isolated anterior table fractures represent a different challenge: restoration of the forehead contour [1,3,4]. Displaced and comminuted anterior table fractures can result in persistent aesthetic deformity because small, thin, and irregular fragments are difficult to mobilize, anatomically reduce, and stabilize using standard osteosynthesis alone [3,4]. When stable anatomical reduction cannot be achieved, titanium mesh reconstruction is often used as a pragmatic alternative to restore contour [5].

Titanium plates and screws remain the reference standard for rigid craniofacial fixation; however, conventional hardware may be suboptimal for small comminuted fragments due to limited screw purchase, fragment fragility, and the need to avoid excessive implant burden [3,4]. In this context, tissue adhesives—especially cyanoacrylate-based glues—have been explored as adjuncts to assist in reduction and stabilize fragments that cannot be reliably secured with screws. Clinical reports in cranio-maxillofacial trauma (e.g., orbital–maxillo–zygomatic and zygomatic tripod fractures) suggest feasibility in selected scenarios [6,7], and a prospective clinical study has evaluated *N*-butyl-2-cyanoacrylate for comminuted fractures of the anterior wall of the maxillary sinus, supporting its potential role in “small-fragment” environments [8]. At the same time, experimental and biomechanical literature underlines that cyanoacrylates should not be considered a replacement for rigid fixation in bone surgery; rather, their role is best conceptualized as a coadjuvant in carefully selected scenarios, given variability in biomechanical performance and ongoing debate regarding healing response and biocompatibility across models and formulations [9,10,11,12]. Broader clinical reviews in dentistry also highlight heterogeneous evidence and emphasize cautious, indication-driven use [13].

In parallel, three-dimensional (3D) virtual surgical planning and additive manufacturing have expanded the surgeon’s ability to manage complex craniofacial defects with improved precision. High-resolution computed tomography enables accurate fracture characterization, and 3D models can facilitate preoperative planning and intraoperative guidance for fragment repositioning and fixation strategy [14]. Importantly, point-of-care (POC) or “in-house” manufacturing workflows have been proposed to reduce lead time and costs, enabling rapid iteration and availability of patient-specific models and guides directly within hospital-based manufacturing units [15]. Recent experiences in academic OMFS environments have further reinforced the feasibility and clinical value of POC manufacturing for patient-specific surgical aids [16,17,18].

Despite these developments, there is limited literature describing an integrated approach that combines POC-manufactured frontal sinus biomodels serving as an extracorporeal reduction template for comminuted anterior table fractures together with cyanoacrylate-assisted stabilization as an adjunct to microplate fixation. Such a strategy may be particularly valuable in isolated anterior table comminution, where the primary objective is restoration of forehead contour while preserving sinus drainage when possible and avoiding more aggressive sinus procedures that are not otherwise indicated [1,2,3,4].

Therefore, this paper aims to describe a reproducible point-of-care workflow for digitally guided stabilization of a comminuted anterior table frontal sinus fracture, leveraging an in-house manufactured biomodel to guide anatomical reconstruction and using a modified *N*-butyl-2-cyanoacrylate adhesive (Glubran2^®^ (GEM S.r.l., Viareggio, Italy)) as a coadjuvant to titanium microplate fixation to maintain reduction of fragments with limited screw purchase.

## 2. Materials and Methods

### 2.1. Study Design

This manuscript reports a case-based technical workflow for the reconstruction of a comminuted anterior table frontal sinus fracture using (i) point-of-care (POC) virtual planning and in-house 3D-printed biomodels as an anatomical reduction template and (ii) cyanoacrylate adhesive (Glubran2^®^) as an adjunct to titanium microplate fixation. The goal was to enable reproducible extracorporeal fragment reassembly (“puzzle” reduction) and stable fixation while preserving sinonasal drainage whenever feasible.

### 2.2. Ethics, Consent, and Privacy

The procedure was performed as part of routine clinical care at Gregorio Marañón Research Institute and Gregorio Marañón University Hospital. Ethical review and approval were not required for this case report because it includes only fully anonymized clinical and radiological data, in accordance with institutional policy and applicable regulations. Written informed consent was obtained from the patient for the surgical procedure and for the publication of anonymized images and radiological findings. All patient data were handled in compliance with institutional and national data-protection regulations.

### 2.3. Patient Assessment and Imaging Acquisition

**Figure 1:**
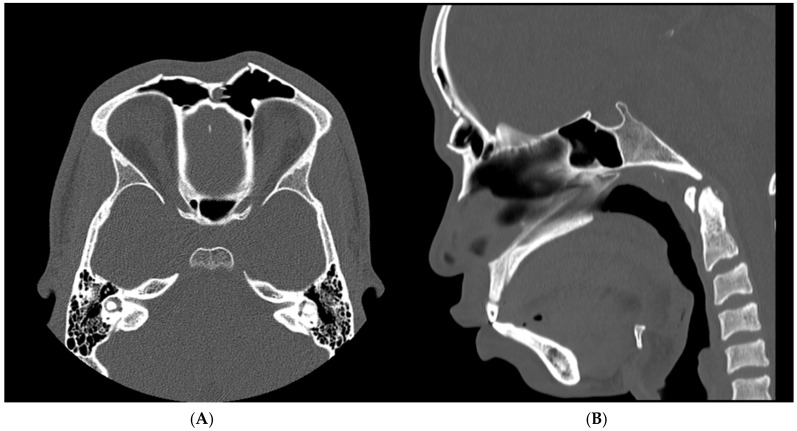
Axial (**A**) and sagittal (**B**) CT views of the anterior wall frontal sinus fracture. The images reveal a significant deformity with disruption of the sinus contour, demonstrating the extent of bony displacement.

A young adult patient sustained blunt frontal trauma (assault) with clinically evident depression of the frontal contour. High-resolution craniofacial CT with thin-slice acquisition was obtained to characterize the fracture pattern, assess anterior/posterior table involvement, and guide management. Imaging confirmed a depressed comminuted anterior table fracture; the surgical plan prioritized restoration of forehead contour and preservation of frontal sinus drainage when possible (preoperative imaging is presented in Section 2; Figure 1).

### 2.4. Digital Planning and 3D Biomodel Design (POC Workflow)

The CT DICOM dataset was imported into medical image-processing software Mimics (version 28.0, Materialise NV, Leuven, Belgium) for segmentation of the frontal bone and fracture components. Bone segmentation was performed using thresholding and region-growing, followed by manual refinement to accurately delineate fracture margins and comminuted fragments. A 3D surface model (STL) was generated and checked for artifacts (holes, non-manifold edges), then minimally smoothed in non-critical regions to preserve anatomical landmarks.

To facilitate reduction and provide intraoperative guidance, two complementary models were designed:

1. Defect/hollow model: reproducing the depressed region and fracture void to improve the three-dimensional understanding of the defect.
2. Reconstruction/template model: representing the intended anatomical anterior table contour to guide extracorporeal reassembly of the comminuted fragments against a stable reference surface.

The STL files were prepared for additive manufacturing (slicing parameters optimized for dimensional accuracy) and 3D printed in-house within a hospital POC manufacturing environment. Post-processing included support removal and surface finishing. Quality control consisted of visual inspection and physical verification to ensure that the comminuted fragments could be accurately positioned against the reconstruction template.

**Figure 2:**
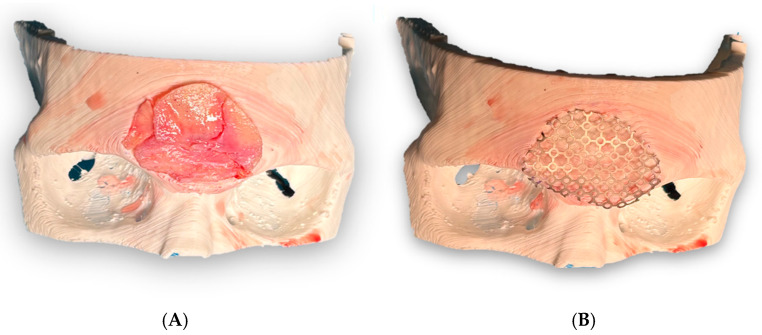
3D-printed model of the fractured frontal sinus used for preoperative and intraoperative planning. (**A**) The comminuted bony fragments were reconfigured extracorporeally to restore anatomical alignment. (**B**) A custom-designed titanium mesh was created based on the 3D model as a backup fixation strategy in case the small, comminuted fragments proved unstable during reconstruction.

As a contingency plan, a patient-adapted titanium mesh was designed based on the 3D model as a backup fixation option should stable reconstruction with comminuted fragments not be feasible (workflow outputs are shown in Section 2; Figure 2).

**Scheme 1:**
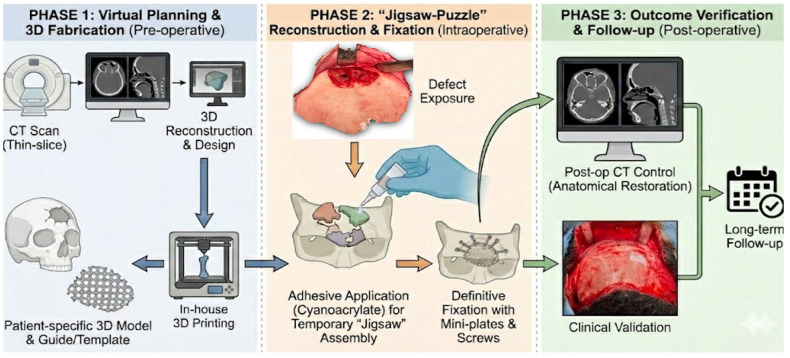
Biomodel-guided “jigsaw-puzzle” reconstruction of a comminuted frontal bone fracture under a point-of-care workflow.

A schematic overview of the biomodel-guided reconstruction protocol is provided in Scheme 1.

Point-of-care biomodel manufacturing was documented through the institutional traceability report. Briefly, segmentation was performed from thin-slice CT data (19 December 2024), generating a 3D model for planning in collaboration with the Oral and Maxillofacial Surgery team. The biomodel was manufactured in-house using fused deposition modeling (FDM) in polylactic acid (PLA). Representative snapshots of segmentation, 3D model generation, and the printed biomodel are provided (Supplementary Figure S1).

### 2.5. Materials (Fixation Systems and Adhesive)

- Adhesive: Glubran2^®^ (GEM S.r.l., Viareggio, Italy), a modified *N*-butyl-2-cyanoacrylate.
- Rigid fixation: titanium microplates and screws (standard cranio-maxillofacial osteosynthesis system), with configuration selected intraoperatively according to fragment size and bone thickness.
- Backup strategy: patient-adapted/custom titanium mesh designed from the digital model (prepared but not used in this case).

### 2.6. Surgical Procedure

All steps were performed under general anesthesia.

#### 2.6.1. Surgical Approach and Exposure

A coronal incision was used to obtain wide exposure of the anterior frontal sinus region. Subgaleal dissection followed by subperiosteal elevation exposed the comminuted anterior table fragments. Fracture fragments were carefully mobilized and removed to permit unobstructed assessment of the fracture bed and to avoid malreduction caused by interposed debris.

#### 2.6.2. Assessment of Frontal Sinus Drainage and Sinus Preservation Strategy

A key intraoperative decision point was the evaluation of the sinonasal drainage pathway. Because the drainage route was confirmed to be intact, sinus obliteration or cranialization was not performed, and reconstruction focused on restoring the anterior table contour.

#### 2.6.3. Extracorporeal Fragment Reassembly Using the 3D Template

Fragments were reconstructed extracorporeally against the 3D reconstruction/template model to restore the premorbid curvature and surface continuity. This “puzzle” step allowed controlled alignment outside the operative field and reduced repeated intraoperative manipulation prior to reimplantation.

#### 2.6.4. Adhesive-Assisted Stabilization and Definitive Fixation

**Figure 3:**
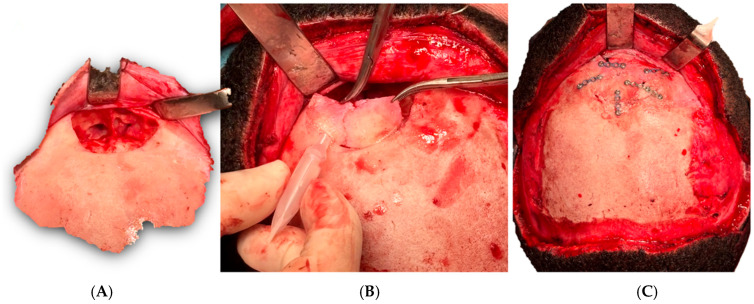
Intraoperative steps of frontal sinus fracture repair. (**A**) The frontal bone defect is exposed, revealing the fractured area and the underlying sinonasal tract. (**B**) Application of surgical adhesive (Glubran 2) to secure the repositioned fragments. Due to the rapid polymerization of the glue, minimal to no movement was allowed after placement to ensure stable fixation. (**C**) Final fixation with titanium microplates, each secured with two screws, providing sufficient stability without the need for additional hardware.

The reconstructed fragment assembly was transferred to the defect site. Due to fragment size and geometry, maintaining alignment with plates and screws alone was potentially unstable. Therefore, Glubran2^®^ was used as an adjunct to maintain interfragmentary relationships during definitive fixation:

- The adhesive was applied in minimal, targeted amounts at selected interfragmentary interfaces.
- The construct was held immobile during polymerization to prevent micro-displacement.
- Titanium microplates were then applied to provide long-term mechanical stability, aiming to achieve stable fixation with limited hardware.
- Representative intraoperative steps are provided in Section 2 (Figure 3).

#### 2.6.5. Closure and Immediate Postoperative Care

After confirming stability and restoration of contour by inspection and palpation, the wound was closed in layers. Surgical drains were not used. Standard postoperative analgesia and wound care were prescribed.

### 2.7. Outcome Measures and Follow-Up Assessment

Outcomes were assessed clinically and radiologically:

- Clinical endpoints: restoration of frontal contour; wound complications (infection, dehiscence, hematoma/seroma); sensory disturbances; and patient-reported satisfaction.
- Radiological endpoints: postoperative CT assessment of anterior table alignment and contour restoration.
- Quantitative endpoint (recommended): objective CT-based metrics can complement visual assessment, including (i) craniometric measurements of anterior table displacement/step-off and (ii) 3D surface deviation analysis (deviation maps) by rigidly registering the postoperative CT to the preoperative virtual plan/template and reporting mean absolute/RMS deviation and the percentage of surface within predefined tolerances (e.g., ±1–2 mm). In this initial case report, outcome assessment was primarily qualitative and these quantitative metrics will be prioritized in future series.
- Follow-up: clinical surveillance for sinonasal symptoms and delayed complications.

For reproducibility, the complete point-of-care workflow—including digital planning, in-house biomodel manufacturing, and the intraoperative fixation strategy—is summarized in Table 1.

### 2.8. Data, Materials, and Protocol Availability

- Data: Anonymized imaging excerpts and intraoperative photographs are provided within the manuscript figures. Full raw DICOM data are not publicly deposited due to patient privacy regulations but may be made available upon reasonable request to the corresponding author, subject to institutional review and data-sharing agreements.
- Materials: The adhesive and fixation systems are commercially available as specified.
- Protocols: The digital planning and POC manufacturing workflow is described step-by-step in Section 2.4, Section 2.5 and Section 2.6 to enable replication.

POC manufacturing and biomodel-based templating can be particularly valuable in comminuted anterior table fractures, where small fragments and loss of surface continuity make in situ reduction challenging. The physical template enables extracorporeal anatomical reassembly and provides an objective reference for restoring the premorbid forehead curvature, while a backup patient-adapted mesh can be planned in parallel to mitigate intraoperative uncertainty. To facilitate reproducibility, the complete point-of-care workflow—including digital planning, in-house biomodel manufacturing, and the intraoperative fixation strategy—is summarized in Table 1.

## 3. Results

### 3.1. Preoperative Findings

Preoperative thin-slice CT demonstrated an isolated, comminuted and depressed fracture of the anterior table of the frontal sinus with disruption of the native frontal contour. Axial and sagittal views confirmed marked anterior wall displacement consistent with the clinically evident forehead depression (Figure 1).

### 3.2. Point-of-Care Planning Output and Intraoperative Feasibility

A point-of-care workflow enabled the generation of 3D-printed biomodels used for both anatomical orientation and extracorporeal reduction planning (Figure 2). According to UPAM3D documentation, segmentation was performed from the CT study dated 19/12/2024, and the biomodel was manufactured in-house by FDM technology using PLA.

The reduction template biomodel allowed a reproducible “target contour” reference, facilitating accurate matching of fragment borders during extracorporeal assembly. In parallel, a custom titanium mesh was designed as a contingency option should fragment stability prove insufficient (Figure 2B), although it was not required intraoperatively.

Intraoperatively, after exposure of the anterior table defect, the comminuted fragments were repositioned and stabilized with targeted Glubran 2 application at selected interfaces to maintain reduction during handling. Definitive stabilization was achieved with titanium microplates, providing a stable construct while limiting the amount of hardware required (Figure 3).

### 3.3. Postoperative Outcomes and Follow-Up

**Figure 4:**
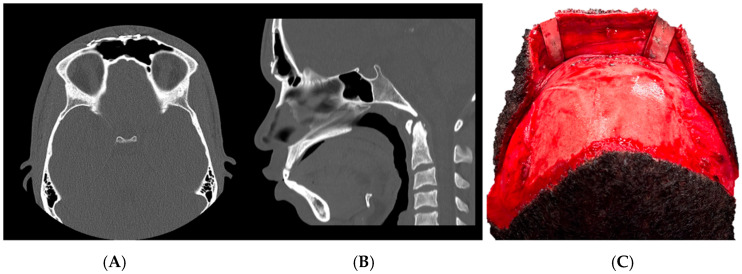
Postoperative evaluation of frontal sinus fracture repair. (**A**) Axial and (**B**) sagittal CT scans demonstrating successful restoration of the frontal sinus contour with proper alignment of the reconstructed fragments. (**C**) Intraoperative view showing the smooth restoration of the forehead contour, confirming anatomical reconstruction and stability of the repaired sinus.

Immediate postoperative assessment showed restoration of the frontal contour with a smooth forehead profile on intraoperative inspection after fixation (Figure 4C). Postoperative CT confirmed anatomical reconstitution of the anterior table contour and appropriate alignment of the reconstructed fragments on axial and sagittal views (Figure 4A,B).

The postoperative course was uneventful, and the patient was discharged the following day without early complications. At 6-month follow-up, the patient remained asymptomatic with no associated symptoms and no reported complications.

## 4. Discussion

Frontal sinus fractures remain challenging injuries because treatment decisions must balance aesthetic contour restoration with sinus-related risks (e.g., mucocele, infection, late contour deformity) and, when present, posterior table or nasofrontal outflow tract involvement. Contemporary algorithms emphasize tailoring the approach to the fracture pattern, the status of the frontonasal drainage pathway, and the degree of comminution, with goals that include stable reconstruction and avoidance of long-term sinonasal sequelae [1,2,3,4]. In the present case, an isolated, comminuted anterior table fracture with intact drainage was managed with a biomodel-guided extracorporeal “jigsaw-puzzle” reduction and cyanoacrylate-assisted stabilization prior to definitive microplate fixation, achieving contour restoration and an uneventful 6-month follow-up (Figure 1, Figure 2, Figure 3 and Figure 4; Scheme 1).

### 4.1. Biosafety Profile of Cyanoacrylate Surgical Adhesives: Rationale, Benefits, and Limitations

The clinical attractiveness of *N*-butyl-2-cyanoacrylate adhesives in surgery is largely driven by rapid polymerization, strong initial bonding, and practical advantages such as reduced suture burden, potential hemostatic assistance, and workflow simplification in selected scenarios [13,19,20,21,22]. However, cyanoacrylates have historically raised biosafety concerns related to local tissue reaction, heat generation during polymerization, and potential cytotoxicity, particularly with earlier formulations and non-medical-grade products [10,23,24,25]. Experimental and translational literature support that tissue response depends on multiple factors: monomer type, volume applied, tissue environment (moisture, blood), and polymerization dynamics [10,23,24,25]. These principles are consistent with mechanistic work characterizing polymerization behavior of *N*-butyl cyanoacrylate in interventional settings [26] and with in vitro testing of Glubran2 mechanical/adhesive properties [27]. From a practical surgical standpoint, the “biosafety” question is, therefore, less about whether cyanoacrylates can be used at all and more about how to apply them safely: minimal volume, targeted interfaces, avoidance of pooling, and respecting polymerization behavior.

In this case, Glubran2^®^ was used as micro-dots at selected interfragmentary interfaces (rather than as a load-bearing bulk layer), with the intent to provide temporary interfragment stabilization during transfer and plating. This strategy aims to leverage the early adhesive benefit while mitigating the risks associated with excessive adhesive mass and thermal or inflammatory effects.

### 4.2. Novelty and Current Evidence Gap in Cranio-Maxillofacial Osteosynthesis

Despite decades of cyanoacrylate use in medicine, the cranio-maxillofacial literature on bone fixation remains limited and heterogeneous, particularly for comminuted fractures of the upper third and frontal sinus region. Available reports range from early experimental assessments of cytotoxicity and tissue effects [23,24,25] to histotoxicity/healing studies in craniofacial bone contexts [11,12,28]. Recent reviews in dentistry and oral surgery describe broad soft-tissue applications and selected bony or membrane-related indications, but they also highlight the variability of evidence and the absence of high-level comparative trials for many maxillofacial fracture settings [13]. Consequently, the present workflow contributes mainly as a technical proof-of-concept: cyanoacrylate is used not as a substitute for plates/screws, but as a coadjuvant tool to preserve and stabilize small fragments during definitive osteosynthesis.

### 4.3. Evidence Suggesting Inferior Osteosynthetic Performance Compared with Screws/Titanium

Several studies indicate that cyanoacrylate adhesives, when used as the primary fixation modality, may have inferior mechanical behavior compared with conventional screw-based fixation—particularly for constructs exposed to functional load or requiring long-term rigid stability. A histomorphologic onlay graft study reported superior outcomes with screw fixation compared with *N*-butyl-2-cyanoacrylate used alone [9], and short-term clinical evaluations of graft fixation have reported comparable concerns [29]. Biomechanical comparisons in craniofacial surgery have also shown that cyanoacrylate glue fixation does not consistently reproduce the stability of plate-and-screw constructs across clinically relevant loading conditions [30,31]. These findings align with the broader principle that, while adhesives can provide meaningful early stabilization, rigid fixation remains the gold standard for long-term mechanical integrity in many craniofacial reconstructions [3,4,5].

This is precisely why, in our approach, cyanoacrylate was intentionally framed as an adjunct rather than a replacement: it functions to hold comminuted segments together long enough to permit effective microplate placement, especially when fragment size or geometry makes direct screw purchase unreliable.

### 4.4. Cyanoacrylate as a Coadjuvant: Where It May Add Value

In comminuted fractures, the main technical failure mode is often not “plate breakage” but fragment management: small fragments are difficult to grasp, align, and maintain while drilling/screwing; they may fracture further, rotate, or become non-reconstructible. Cyanoacrylate can address this specific problem by acting as a temporary “reduction stabilizer”, preserving interfragment relationships during manipulation and plating. Similar coadjuvant logic is reflected in other maxillofacial contexts where adhesives have been used to facilitate closure, reduce operating time, or stabilize tissues [19,20,21,22,32]. In selected fracture settings, clinical series support feasibility in thin bony regions, such as the orbital–maxillo–zygomatic complex and tripod fractures [6,7]. The prospective study on comminuted anterior wall maxillary sinus fractures further supports interest in adhesives for comminution-dominant scenarios [8]. Additionally, cyanoacrylates have been used to manage sinus membrane perforations in sinus-lift procedures, illustrating their utility in delicate sinonasal-adjacent tissues [33].

Within the upper third, titanium mesh reconstruction has been reported as a practical solution for comminuted anterior table fractures when fragment salvage is not feasible or predictable [5]. Our workflow preserves this concept as a backup plan (Figure 2B) but prioritizes native fragment reconstruction supported by a biomodel template and adhesive-assisted transfer. From a reconstructive standpoint, fragment-preserving (“biological”) reconstruction may offer advantages over primary alloplastic reconstruction in carefully selected isolated anterior table injuries: it restores the native cortical thickness and curvature using autologous bone, minimizes permanent implant burden in a thin forehead soft-tissue envelope (potentially improving long-term palpability and “feel”), and preserves the possibility of osseous union and remodeling at the fracture lines. Titanium mesh remains an effective and pragmatic option when fragments are missing, non-viable, or cannot be stably reduced, but it represents a larger permanent alloplastic surface area and may be associated with implant-related palpability or exposure in some patients.

### 4.5. Comparative Perspective by Cranio-Maxillofacial Subsite

The cranio-maxillofacial skeleton is not mechanically uniform, and the applicability of adhesives differs by region:

- Mandible (load-bearing): High functional loads and micromotion make adhesive-only fixation less attractive. Mandibular studies (pilot clinical and osteotomy fixation) illustrate feasibility but reinforce that rigid stability remains critical, and adhesive-only constructs should be interpreted cautiously [34,35].
- Midface (variable load): The maxilla and zygoma require precise 3D alignment and experience complex force vectors. Reports of cyanoacrylate use in zygomatic or orbital–maxillo–zygomatic fractures suggest feasibility in selected cases, but evidence remains limited [6,7].
- Upper third/frontal sinus anterior table (non-load-bearing contour unit): This region is primarily contour-bearing rather than masticatory load-bearing. Key endpoints are contour restoration, stable bony union, and avoidance of sinus complications. In this setting, cyanoacrylate as a coadjuvant is most defensible: it can stabilize small segments without extensive hardware, potentially reducing palpability while rigid fixation provides definitive stability [1,2,3,4,5].

### 4.6. Expected Behavior in Load-Bearing vs. Non-Load-Bearing Facial Bone

A useful conceptual distinction is whether the reconstructed segment is load-bearing (mandible; to some extent, the buttress system) versus non-load-bearing (anterior table contour segment). Adhesive-only constructs are more likely to fail under cyclical load or when prolonged rigidity is required; conversely, in non-load-bearing contour units, the adhesive’s role can be limited to temporary stabilization, with microplates ensuring the final mechanical construct. This rationale is consistent with comparative biomechanical studies [30,31,36] and with craniofacial healing/histotoxicity observations that support careful, indication-driven use [11,12,28,37,38,39].

### 4.7. Technical Novelty: Extracorporeal Reduction Guided by POC Biomodel and Academic Manufacturing

A second key innovation in this report is the workflow design rather than any single material: biomodel-guided extracorporeal “jigsaw-puzzle” reduction performed under a hospital point-of-care manufacturing pathway (Scheme 1). “Jigsaw-puzzle” reconstruction using 3D printing has been described for comminuted craniomaxillofacial fractures, demonstrating how physical models can improve fragment repositioning and surgical efficiency [14]. Our adaptation emphasizes the use of a reduction template biomodel as a true anatomical contour reference, combined with adhesive-assisted transfer to maintain the extracorporeally achieved assembly during reimplantation and plating.

POC manufacturing and biomodel-based templating may be particularly valuable in comminuted anterior table fractures, where small fragments and loss of surface continuity make in situ reduction challenging. In this context, the physical template facilitates extracorporeal anatomical reassembly and provides an objective reference for restoring the premorbid forehead curvature, while a backup patient-adapted mesh can be planned in parallel to reduce intraoperative uncertainty. More broadly, this approach aligns with the expanding role of academic point-of-care manufacturing in oral and maxillofacial surgery, where in-house workflows can shorten iteration cycles, increase surgeon control over the plan-to-execution chain, and enable patient-specific aids such as models, guides, and contingency implants [15,16,17,18]. In this case, the backup mesh design illustrates a pragmatic hybrid strategy: fragment-preserving reconstruction when possible, with a preplanned alloplastic alternative available if intraoperative feasibility proves inadequate.

### 4.8. Future Directions

Several research directions emerge from this experience:

1. Standardized protocols for adhesive use in craniofacial fracture fixation, including recommended volumes, application sites, and precautions based on polymerization behavior and tissue environment [26,27].
2. Comparative biomechanical testing of “microplate-only” versus “adhesive + microplate” constructs for comminuted anterior table models, focusing on resistance to displacement and fragment loss rather than gross load-bearing strength [30,31].
3. Prospective multicenter registries of adhesive-assisted fixation in cranio-maxillofacial trauma, capturing outcomes relevant to the frontal sinus (contour, sinus complications, revision rate) [1,2].
4. POC workflow evaluation (time, cost, decision impact): assessing whether biomodel-guided extracorporeal reduction reduces operative time or hardware requirements compared with conventional in situ reduction [14,15,16,17,18].
5. Longer follow-up to monitor late sinus-related sequelae (e.g., mucoceles), which remain central concerns in frontal sinus trauma management [1,2,4].

### 4.9. Limitations and Need for Validation

This report has limitations typical of single-patient technical notes. First, external validity is limited, and the protocol should be considered applicable only to carefully selected fractures (isolated anterior table comminution with preserved frontal sinus drainage and no CSF leak) where fragments can be mobilized and reassembled without compromising tissue viability.

Second, a 6-month follow-up is short for the frontal sinus region. Because mucoceles, chronic infection/osteomyelitis, and other sinonasal sequelae may manifest years after injury or intervention, long-term surveillance is essential and should extend beyond 24 months and ideally several years, consistent with contemporary frontal sinus fracture management principles [1,2,4].

Third, outcome assessment in this initial report is primarily qualitative. Future work should incorporate objective quantitative metrics, such as 3D surface deviation maps comparing the preoperative virtual plan/template with the postoperative CT and/or standardized CT craniometric measurements of displacement and angulation. Finally, while the workflow is designed to be reproducible, point-of-care manufacturing requires dedicated infrastructure, traceability, and governance; therefore, multicenter cohorts and/or registries are needed to establish reproducibility, define indications, and assess long-term sinonasal outcomes.

## 5. Conclusions

This technical report describes a biomodel-guided “jigsaw-puzzle” workflow for comminuted anterior table frontal sinus fracture reconstruction, integrating point-of-care virtual planning and academic in-house manufacturing of 3D-printed biomodels with cyanoacrylate (Glubran2^®^)-assisted fragment stabilization and definitive titanium microplate fixation.

Beyond improving anatomical understanding, the availability of a sterile, immediately accessible biomodel/template produced under a point-of-care pathway provided intraoperative optionality: it enabled extracorporeal fragment reassembly when in situ reduction was impractical and supported rapid decision-making between alternative fixation strategies (native fragment salvage vs. preplanned backup solutions). In this context, POC academic manufacturing acts as an intraoperative “decision amplifier”, expanding reconstructive possibilities without delaying care.

The approach achieved restoration of frontal contour with radiological confirmation and an uncomplicated 6-month follow-up, while preserving frontal sinus drainage when intact. Cyanoacrylate appears most valuable as a coadjuvant—facilitating temporary stabilization of small, irregular fragments and transfer of the reconstructed assembly—rather than as a substitute for rigid fixation.

Larger series and comparative studies are warranted to define indications, reproducible technical parameters (template design, adhesive volume/application points), cost–time impact of point-of-care manufacturing, and long-term sinonasal outcomes in frontal sinus fracture management.

Given the single-case nature of this report and the limited follow-up, the findings should be interpreted as preliminary. Long-term clinical and radiological surveillance (at least >2 years) is recommended for frontal sinus fractures to detect delayed sinonasal complications, and future multicenter cohorts incorporating quantitative 3D outcome metrics will be necessary to establish the reliability, indications, and comparative value of this protocol.

## Figures and Tables

**Table 1:** Point-of-care (POC) digital workflow for comminuted anterior table frontal sinus fracture reconstruction: steps, outputs, and clinical endpoints.

Workflow Step	Key Actions (Replicable Details)	Output(s)	Clinical Endpoint(S)/Decision Points
1. Clinical assessment	CMF trauma evaluation; inspection/palpation of forehead depression; surgical indication based on deformity and fracture pattern	Clinical baseline	Indication for anatomical contour restoration and stable fixation
2. CT imaging acquisition	High-resolution craniofacial CT with thin-slice protocol	DICOM dataset	Define fracture pattern; assess anterior/posterior table involvement; plan sinus preservation strategy
3. Image segmentation	Thresholding + region growing; manual refinement of fracture margins; artifact correction	3D bone/fracture segmentation	Accurate delineation of comminuted fragments for planning and modeling
4. 3D model generation	STL creation; mesh repair (holes/non-manifold); minimal smoothing preserving landmarks	Printable STL model	Reliable geometric substrate for template design and intraop guidance
5. Biomodel design	Two-part approach: (i) defect/hollow model; (ii) reconstruction/template model representing intended premorbid contour	Defect model + anatomical reconstruction template	Enable extracorporeal “puzzle” reduction; reduce intraoperative guesswork
6. Backup fixation planning	Design of patient-adapted titanium mesh from digital model (contingency strategy)	Backup mesh design	Decision point if fragment viability/stability is insufficient for reconstruction
7. POC manufacturing	In-house 3D printing; post-processing (supports removal/finishing); dimensional QC (visual inspection + fitting)	Physical biomodel(s) available for planning/intraop reference	Improve reduction accuracy and efficiency; provide tangible contour reference in comminution
8. Surgical exposure	Coronal approach; subgaleal/subperiosteal elevation; careful fragment mobilization/removal; bed preparation	Exposed defect and free fragments	Optimize conditions for precise reduction; avoid malreduction by debris/interposed bone
9. Sinus drainage assessment	Intraoperative evaluation of sinonasal drainage pathway	Drainage status (intact vs. compromised)	Decision: preserve sinus vs. obliterate/cranialize; in this case, preservation
10. Extracorporeal reduction (“puzzle”)	Fragment reassembly against reconstruction template outside operative field	Reassembled fragment construct	Reproduce anatomical curvature; minimize repeated in-field manipulation
11. Adhesive-assisted stabilization	Targeted, minimal Glubran2^®^ at selected interfaces; immobilization during polymerization	Temporary interfragmentary stability	Maintain alignment of small/irregular fragments that are difficult to screw/plate alone
12. Definitive rigid fixation	Titanium microplates/screws placed to reinforce construct with limited hardware	Stable osteosynthesis	Long-term mechanical stability; minimize palpability/hardware burden
13. Closure and immediate care	Layered closure; no drain; routine analgesia/wound care	Uneventful early recovery	Early endpoint: absence of wound issues/CSF leak/sinonasal symptoms
14. Postoperative imaging	CT to assess contour restoration and fragment alignment	Postoperative CT confirmation	Radiological endpoint: restored anterior table contour and alignment
15. Follow-up	Clinical surveillance including sinonasal symptoms and signs of delayed complications	6-month clinical follow-up	Endpoint: asymptomatic course; no infection, instability, or sinus-related complaints

## Data Availability

Data supporting the findings of this study are available within the article. Additional anonymized data may be available from the corresponding author upon reasonable request, subject to institutional requirements and patient privacy regulations.

## Supplementary Materials

The following supporting information can be downloaded at: https://www.mdpi.com/article/10.3390/jcm15052057/s1, Figure S1: Segmentation-to-print workflow for the frontal sinus biomodel (point-of-care manufacturing).
