# Supplementary material for: Digitally Guided Frontal Sinus Fracture Fixation: A Point-of-Care “In-House” Biomodel Protocol with Cyanoacrylate-Assisted Fragment Stabilization
Source: J Clin Med. 2026 Mar 8;15(5):2057. doi: 10.3390/jcm15052057 (PMC12986275; doi:10.3390/jcm15052057)

Figure S1. Segmentation-to-print workflow for the frontal sinus biomodel (point-of-care manufacturing).

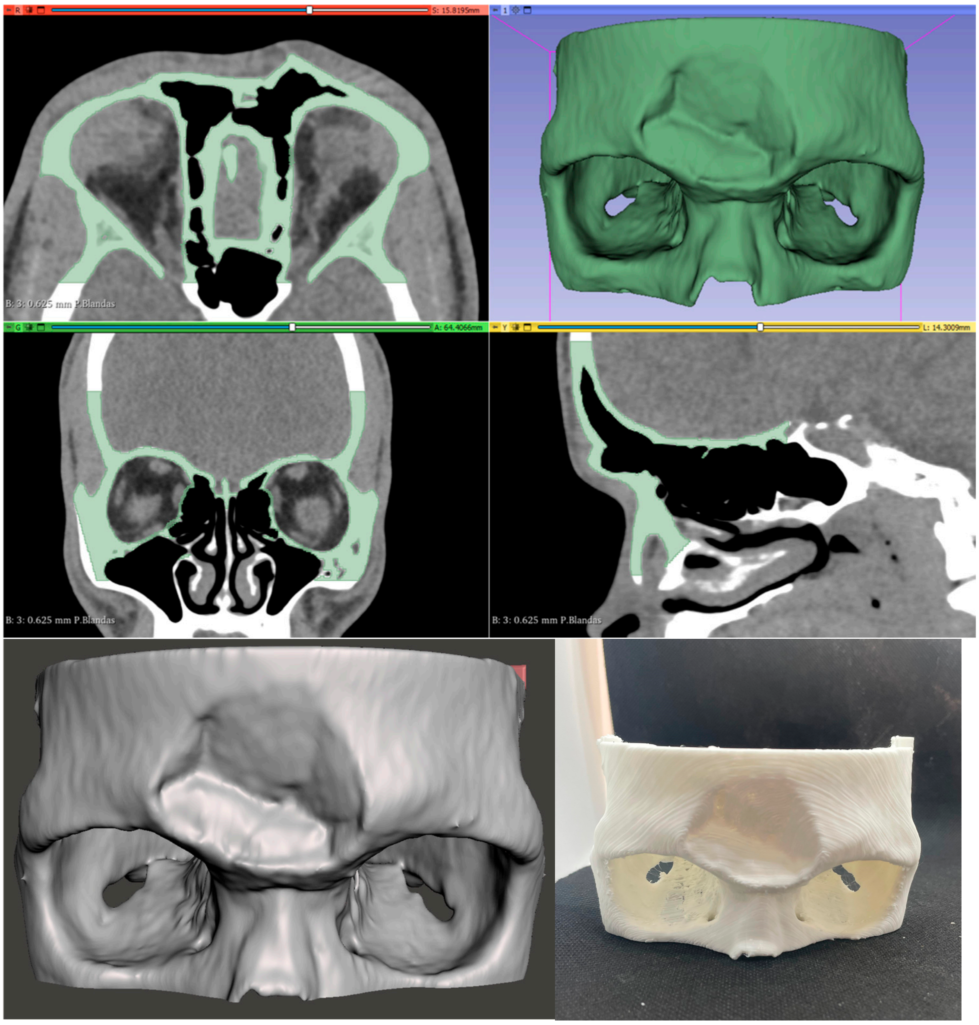

Supplement: Supplementary file 1 [file jcm-15-02057-s001.zip › jcm-4189653-supplementary.pdf]
